# Associations of maternal resources with care behaviours differ by resource and behaviour

**DOI:** 10.1111/mcn.12977

**Published:** 2020-03-26

**Authors:** Sulochana Basnet, Edward A. Frongillo, Phuong Hong Nguyen, Spencer Moore, Mandana Arabi

**Affiliations:** ^1^ Department of Health Promotion, Education, and Behavior University of South Carolina Columbia South Carolina USA; ^2^ Poverty, Health, and Nutrition Division International Food Policy Research Institute Washington DC USA; ^3^ Global Technical Services Nutrition International Ottawa Canada

**Keywords:** care behaviours, child feeding, family care behaviours, hygiene practices, immunization, resources for care

## Abstract

Care is important for children's growth and development, but lack or inadequacy of resources for care can constrain appropriate caregiving. The objectives of this study were to examine whether maternal resources for care are associated with care behaviours specifically infant and young child feeding, hygiene, health‐seeking, and family care behaviours. The study also examined if some resources for care are more important than others. This study used baseline Alive & Thrive household surveys from Bangladesh, Vietnam, and Ethiopia. Measures of resources for care were maternal education, knowledge, height, nourishment, mental well‐being, decision‐making autonomy, employment, support in chores, and perceived instrumental support. Multiple regression analyses were conducted to examine the associations of resources for care with child‐feeding practices (exclusive breastfeeding, minimum meal frequency, dietary and diversity), hygiene practices (improved drinking water source, improved sanitation, and cleanliness), health‐seeking (full immunization), and family care (psychosocial stimulation and availability of adequate caregiver). The models were adjusted for covariates at child, parents, and household levels and accounted for geographic clustering. All measures of resources for care had positive associations with care behaviours; in a few instances, however, the associations between the resources for care and care behaviours were in the negative direction. Improving education, knowledge, nutritional status, mental well‐being, autonomy, and social support among mothers would facilitate provision of optimal care for children.

**Key Messages**

- Previous studies indicate that the provision of optimal care is important for improving child growth, development, and survival; however, lack of resources for care among caregivers especially mothers may constrain optimal care behaviours.
- Maternal education, knowledge, nutritional status, psychological well‐being, autonomy, and social support are important for improved child feeding, hygiene, immunization, and family care behaviours.
- The associations of maternal care resources with care behaviours differed by the type of resource and care behaviour.
- Future programmes and policies targeting to improve care received by children must strengthen resources for care available to mothers.

## 1 INTRODUCTION

Care behaviours are crucial for children's optimal growth, development, and survival (Engle, Menon, & Haddad, 1999). Care received by children has positive impacts on later adult health and productivity (Britto et al., 2017; Engle et al., 1999). Care refers to the behaviours that provide time, attention, and support to family members including children to meet physical and psychosocial needs (Engle, 1999; Engle et al., 1999). Both provision and quality of care are critical to children's health and well‐being (Engle, 1999). Appropriate care can mitigate or prevent adverse consequences of unfavourable conditions such as poverty, violence, and chronic disease (Britto et al., 2017; Britto & Engle, 2015; Purcell‐Gates, 1996). Despite the increasing recognition of importance of care behaviours, substantial number of children do not receive optimal care (Bhutta et al., 2008; Black et al., 2017; Engle et al., 1999) such as optimal feeding (Arabi, Frongillo, Avula, & Mangasaryan, 2012), hygiene (Rah et al., 2015), health care (Haji et al., 2016), and psychosocial stimulation (Bornstein & Putnick, 2012).

The multiple determinants of care behaviours range from social contexts at home to national policies and programmes (Britto et al., 2017; Frongillo, Kulkarni, Basnet, & de Castro, 2017). Family represent the first environment that a child experiences right after birth, and it also reflects the environment in which a child spends most periods of life that are sensitive for growth and development (Frongillo et al., 2017; Maggi, Irwin, Siddiqi, & Hertzman, 2010). Therefore, availability of resources to caregivers could be crucial in determining care behaviours.

Resources for care enhance caregiver's capabilities in meeting mental, physical, and social needs of household members including children (Engle et al., 1999; Engle, Menon, & Haddad, 1997). Theoretically, caregiver's education and knowledge, physical health, mental well‐being, autonomy, reasonable workload/availability of time, and social support are important to provide care (Engle et al., 1997; Engle et al., 1999; Peter & Kumar, 2014). Education and knowledge indicate the capacities of caregivers for providing care. Higher education and knowledge also help to develop positive attitude toward care and that may facilitate translation of acquired knowledge in order to improve care practices (Engle et al., 1999). Physical and mental health represent individual‐level conditions that help in the translation of capacities to behaviours. Autonomy indicates the ability of caregivers to have a control of their surroundings (Carlson, Kordas, & Murray‐Kolb, 2015; Engle et al., 1999). Reasonable workload and social support are the conditions related to family and community that help in improving caregiver's ability to provide care (Engle et al., 1999). In many societies around the world, mothers are primary caregivers, and resources available to them may play a pivotal role on determining care behaviours (Coller & Kuo, 2015; Engle et al., 1999). Understanding the role of care resources among mothers on care received by children could be helpful in implementing policies and interventions that aim to improve care behaviours (Peter & Kumar, 2014).

Associations of maternal resources for care with care behaviours have been investigated, yet some types of resources and care behaviours have been studied more extensively than others (Engle et al., 1999). Additionally, there is a dearth of research that has examined all categories of resources for care and multiple care behaviours. Some studies have shown inconsistent findings on the associations of maternal resources with care behaviours. For example, poor maternal mental well‐being was a risk factor for decreased self‐efficacy regarding breastfeeding, difficulty in breastfeeding, and decreased breastfeeding duration in a qualitative review (Dennis & McQueen, 2009). In contrast, a study by Chung et al. reported no association between maternal mental health and likelihood of breastfeeding for ≥1 month (Chung, McCollum, Elo, Lee, & Culhane, 2004). These inconsistent findings warrant further investigation about the role of maternal resources for care on improving care behaviours.

The objective of the present study was to examine whether maternal resources for care are associated with care behaviours, specifically infant and young child feeding (IYCF) practices, hygiene practices, health‐seeking practice, and family care behaviours in Bangladesh, Vietnam, and Ethiopia. This study also investigated if some resources for care are more important for care behaviours than other resources for care.

## 2 METHODS

### 2.1 Data source and study participants

We used the Alive & Thrive baseline data that were collected in Bangladesh, Vietnam, and Ethiopia in 2010. Alive & Thrive is an initiative that intends to have positive impact on children's survival, growth, and development by improving IYCF practices (Nguyen et al., 2017). In Bangladesh, households were selected from 20 subdistricts (*upazilas*). In Vietnam, households were selected from 40 communes representing four provinces. In Ethiopia, households were selected from 75 enumeration areas in two regions. Mothers and their <5 years old children were included in the surveys. Details on sampling procedure can be found elsewhere (Ali et al., 2011; Nguyen et al., 2010; Saha et al., 2011). In the present study, data pertaining to mothers and index/youngest children were used (Bangladesh *n* = 4,400, Vietnam *n* = 4,029, and Ethiopia *n* = 2,746). The surveys received ethical approval from the institutional review board of each country and the International Food Policy Research Institute.

### 2.2 MEASURES

#### 2.2.1 Care behaviours

We used four domains of care behaviours: IYCF, hygiene, health‐seeking, and family care. The IYCF measures were exclusive breastfeeding (EBF; proportion of 0–5.9 months old infants who are exclusively breastfed); minimum meal frequency (proportion of 6–23.9 months old children who receive solid or semi‐solid foods at least minimum number of times, i.e., two times for breastfed 6–8 months old, three times for breastfed 9–23 months old, four times for non‐breastfed 6‐23 month old); and dietary diversity scores (number of food groups consumed by 6–23.9 months old children; range: 0–7; World Health Organization, 2008). The information related to IYCF practices was based on mothers' recall within the past 24 hr.

Improved drinking water source, improved sanitation, and cleanliness were used as measures of hygiene practices. Improved sources of drinking water are those that are adequately protected from contamination like faeces (Pullan, Freeman, Gething, & Brooker, 2014; World Health Organization, 2019). Improved sanitation denotes the toilets that prevent human urine and faeces from human contact (Pullan et al., 2014; World Health Organization, 2019). Both improved water and sanitation variables were binary (improved vs unimproved). The cleanliness variable was developed using hygiene spot check data of house interior, house exterior, child's face, child's hands, child's hair, child's cloth/body, mother's face, mother's hands, mother's hair, and mother's clothing. One point was assigned for each observation indicating “clean,” and then the scores were summed to create the cleanliness variable (total possible score of 10).

Child immunization status represented health‐seeking practice. A child ≥12 months was considered fully immunized if he/she had received essential vaccines such as Bacillus Calmette‐Guérin vaccine, Diphtheria‐Pertusis‐Tetanus vaccine, polio vaccine, hepatitis B vaccine, and measles vaccine (Animaw, Taye, Merdekios, Tilahun, & Ayele, 2014; Oyo‐Ita, Nwachukwu, Oringanje, & Meremikwu, 2012; Uddin, Koehlmoos, Saha, & Khan, 2010). Information on immunizations were collected from immunization cards or mother's reporting.

We used two measures of family care behaviours: psychosocial stimulation and availability of adequate caregiver (Frongillo et al., 2017; Kariger et al., 2012). Psychosocial stimulation (range: 0–6) measures number of learning and school‐readiness promoting activities in which an adult is engaged with a child in past 3 days. The activities were (a) reading books, (b) telling stories, (c) singing songs, (d) taking outside, (e) playing, and (f) naming, counting, and drawing things. Information on stimulation was based on mother's reporting and was available only for Bangladesh and Vietnam. Availability of adequate caregiver was defined as not leaving a child alone or with a minor for more than one hour.

#### 2.2.2 Maternal resources for care

Our measures of resources for care were maternal educational attainment, knowledge, height, nourishment, mental well‐being, decision‐making autonomy, employment, support in chores, and perceived instrumental support. Education levels differed across our study settings; therefore, different cut‐offs were used based on specific context (Nguyen, Saha, et al., 2014). No schooling, 1–5 years of schooling, 6–9 years of schooling, and 10–12 years of schooling were the categories in Bangladesh and Ethiopia. In Vietnam, 1–5 years of schooling, 6–9 years of schooling, and 10–12 years of schooling and college or higher were the categories. In all three countries, 1–5 years of schooling was the reference group. Maternal knowledge was specific to the knowledge of the mothers on breastfeeding, complementary feeding, iron deficiency symptoms, vitamin A sources, iodine fortification, food diversity, and hand washing. A point was assigned when a mother provided a correct response, and then the scores were added to create a composite score of maternal knowledge (total possible score of 22 for Bangladesh and 23 for Ethiopia and Vietnam).

Height and nourishment were used as the measures of physical health. Nourishment was categorized as well‐nourished (body mass index of ≥18.5 kg/m^2^) and underweight (body mass index of <18.5 kg/m^2^; World Health Organization, 2016). Mental well‐being was assessed by using the 20‐itemed Self‐Reporting Questionnaire‐20 (SRQ‐20). The SRQ‐20 is a valid and reliable tool to screen mental health in low‐ and middle‐income countries (World Health Organization, 1994). The tool assesses psychological and somatic symptoms by asking mothers whether they had presence of the symptoms in the past 4 weeks. One point was assigned if mothers indicated the absence of a symptom, and then a composite score on mental well‐being was developed with the total possible score of 20.

Decision‐making autonomy reflected involvement of mothers in household decisions about major purchases, cooking, visiting family/friends/relatives, health care visit, family planning, and care of children. A composite score was developed by assigning a point for each response that indicated involvement of mothers in decision‐making alone or along with spouse (total possible score of 11). Maternal employment status (employed vs. not employed) was used as another measure of autonomy.

Support in household chores and perceived instrumental support were used as the measures of social support. Support in chores was created by assigning 1 point for each response that indicated receiving support in chores and addition of the points (total possible scores: 8 for Bangladesh and 9 for Ethiopia and Vietnam). Perceived instrumental support was developed from three items denoting potential help with accommodation, money, and food (total possible score of 3).

#### 2.2.3 Covariates

Child level covariates were child's age and gender. Father's occupation was also used as one of the covariates. The covariates at the household level were household wealth and the number of <5 years old children in the household. Household wealth index was constructed using principal component analysis by including the variables on house and land ownership, quality of house, access to services in the household (for example, electricity, and cooking fuel), and household assets. Component scores obtained from the first component were used to reflect the household wealth.

### 2.3 Statistical analysis

**Figure 1:**
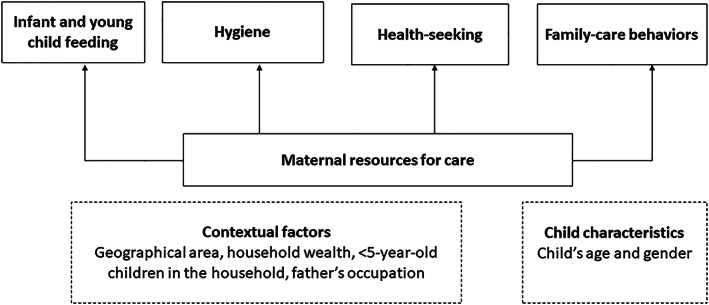
Conceptual framework depicting the role of maternal resources for care on care behaviors

Separate analyses were run for each country using Stata version 14. Sample characteristics were presented as mean and standard deviation (*SD*), or percentage. Multiple regression analyses were performed to examine the associations of resources for care with EBF (children <6 months), complementary feeding (children 6–23.9 months), hygiene practices (children 0–47.9/0–59.9 months), full immunization (children 12–47.9/12–59.9 months), and family care behaviours (children 6–23.9 and children 24–47.9/59.9 months; Figure 1). The analysis for family care behaviours was broken into two age groups because previous studies have suggested that the provision of family care like psychosocial stimulation by the caregivers may differ by child's age (Larson, Martorell, & Bauer, 2018). Additionally, the family care behaviours measured may not present much variation in young children (i.e., <6 months); therefore, this age group was not included in analysis for family care behaviours (Engle et al., 1999; Larson et al., 2018). The selection of age group was guided by previous research and recommendations (Animaw et al., 2014; Uddin et al., 2010; World Health Organization, 2008).

Separate regression models were run for each measure of care behaviour. Each model included all nine measures of resources for care. The models were adjusted for child's age and gender, father's occupation, household wealth, and number of <5 years children in the household. Geographic clustering was accounted using the primary sampling units as random effects. When the magnitude of association was small, we reported odds ratios (OR) or coefficients for a 1 *SD* higher resources for care.

### 2.4 Ethical considerations

This study used the de‐identified data from the Alive & Thrive baseline surveys. The surveys received ethical approval from the institutional review board of each country included in this study and the International Food Policy Research Institute.

## 3 RESULTS

### 3.1 Sample characteristics

The prevalence of maternal 6–9 years of schooling and 10–12 years of schooling was higher in Vietnam than other two countries (Table 1). Mean of maternal knowledge scores were 9.76, 9.01, and 8.88 in Bangladesh, Vietnam, and Ethiopia, respectively. More than 70% of mothers were well‐nourished in all three countries. The mean of maternal height, mental well‐being, and decision‐making autonomy was higher in Ethiopia and Vietnam than Bangladesh. The prevalence of maternal employment also differed across countries with highest prevalence in Vietnam (~90%). Support in chores had means of 4.48, 4.73, and 3.13 in Bangladesh, Vietnam, and Ethiopia, respectively. Perceived instrumental support was higher in Bangladesh and Vietnam than Ethiopia.

**Table 1:** Selected sample characteristics in Bangladesh, Vietnam, and Ethiopia

	Bangladesh (*n* = 4,400)	Vietnam (*n* = 4,029)	Ethiopia (*n* = 2,746)
	Percent or mean ± *SD*		
Maternal characteristics			
Education, %			
No schooling	26.7	—	64.9
1–5 years schooling	29.1	15.7	24.5
6–9 years schooling	36.6	51.5	8.40
10–12 years schooling	7.60	20.4	2.20
College or higher	—	12.4	—
Knowledge (range: B = 0–22, V and E = 0–23)	9.76 ± 2.24	9.01 ± 3.08	8.88 ± 2.97
Height, cm	151 ± 5.51	153 ± 5.26	157 ± 6.04
Well‐nourished, %	70.6	73.5	75.4
Mental well‐being (range: 0–20)	13.1 ± 5.19	15.1 ± 4.50	14.2 ± 4.96
Decision‐making (range: 0–11)	8.33 ± 2.69	9.06 ± 2.03	9.12 ± 2.59
Employed, %	6.20	89.2	48.2
Support in chores (range: B = 0–8, V and E = 0–9)	4.48 ± 3.04	4.73 ± 3.09	3.13 ± 3.47
Perceived instrumental support (Range: 0‐3)	2.73 ± 0.761	2.52 ± 0.809	1.09 ± 1.22	
Child characteristics			
Age, months	22.3 ± 14.4	24.7 ± 17.8	22.8 ± 16.5
Female, %	48.4	47.3	48.5
Household characteristic			
Number of <5 years children, %			
1 child	81.7	81.8	62.6
≥2 children	18.3	18.2	37.4
Father's characteristic			
Occupation, %			
Unemployed/no husband	2.27	1.00	6.88
Farmer	21.1	45.5	87.2
Manual or wage‐based work	32.8	12.9	3.35
Service or salaried work	15.1	11.5	—
Business, trade, or self‐employed	24.3	29.1	2.57
Others	4.43	—	—

*Note.* B = Bangladesh, V = Vietnam, E = Ethiopia.

Child characteristics such as age and gender were similar across three countries. Most of the fathers were engaged in manual or wage‐based work in Bangladesh, whereas as most of the fathers were farmers in Vietnam and Ethiopia.

### 3.2 Care behaviours

Exclusive breastfeeding was higher among infants from Ethiopia (72%) than Bangladesh (50%) and Vietnam (18%; Table 2). Children from Vietnam received better complementary feedings than their counterparts, with about 81% children meeting the minimum meal frequency and mean dietary diversity score of 4.40. Prevalence of improved drinking water and improved sanitation was higher in Bangladesh and Vietnam, respectively, as compared with their counterparts. Additionally, cleanliness score was higher in Bangladesh and Vietnam than Ethiopia. Prevalence of child full immunization was highest in Vietnam, followed by Bangladesh and then Ethiopia. In both Bangladesh and Vietnam, psychosocial stimulation was higher among the older children (24–47.9/59.9 months) than the younger ones (6–23.9 months). Nearly all children in Bangladesh and Vietnam had an adequate caregiver available, whereas in Ethiopia, adequate caregivers were available to only about 69% and 64% of younger and older children, respectively.

**Table 2:** Prevalence or mean of care behaviours in Bangladesh, Vietnam, and Ethiopia

	Bangladesh^f^	Vietnam^g^	Ethiopia^g^
	Percent or mean ± *SD*		
Infant and young child feeding practices			
Exclusive breastfeeding^a^, %	49.9	18.4	72.4
Minimum meal frequency^b^, %	39.6	80.9	45.8
Dietary diversity score^b^ (range: 0–7)	2.85 ± 1.59	4.40 ± 1.59	1.71 ± 1.11
Hygiene practices			
Improved drinking water source^c^, %	97.1	79.2	60.0
Improved sanitation^c^, %	12.3	80.7	1.06
Cleanliness^c^ (range: 0–10)	7.46 ± 2.91	7.13 ± 2.31	5.95 ± 3.09
Health‐seeking practice			
Immunization^d^, %	83.8	84.9	61.2
Family care behaviours			
Psychosocial stimulation in younger children^b^ (range: 0–6), %	2.80 ± 1.58	2.36 ± 1.52	—
Psychosocial stimulation in older children^e^ (range: 0–6)	3.67 ± 1.70	2.51 ± 1.98	—
Adequate caregiver (younger children)^b^, %	97.0	96.3	69.1
Adequate caregiver (older children)^e^, %	96.1	93.4	64.4

a 0–5.9 months.

b 6–23.9 months.

c 0–47.9/0‐59.9 months.

d 12–47.9/12–59.9 months.

e 24–47.9/24–59.9 months.

f Survey included 0–47.9 months old

g Surveys included 0–59.9 months old.

### 3.3 Associations between maternal resources for care and care behaviours

#### 3.3.1 Infant and young child feeding practices

Education was positively associated with EBF in Ethiopia (6–9 years of schooling: OR = 2.80; Table 3). Additionally, education was positively associated with minimum meal frequency (college or higher: OR = 2.34) and dietary diversity in Vietnam (6–9 years of schooling: β = 0.36, college or higher: β = 0.49). Knowledge was positively associated with EBF in Bangladesh and Vietnam, and with dietary diversity in all three countries. Well‐nourishment and height were positively associated with EBF in Bangladesh (OR = 1.60) and Vietnam (OR = 1.26), respectively. Mental well‐being was positively associated with EBF in Bangladesh (OR = 1.21) and Ethiopia (OR = 1.24). Decision‐making autonomy and employment were positively associated with dietary diversity (Vietnam) and minimum meal frequency (Bangladesh), respectively. Support in chores was positively associated with minimum meal frequency in Ethiopia and dietary diversity in Bangladesh and Ethiopia. Additionally, perceived instrumental support was positively associated with dietary diversity in Vietnam and Ethiopia.

**Table 3:** Adjusted associations between maternal resources for care and infant and young child feeding practices

Resources for care	Exclusive breastfeeding (OR)			Minimum meal frequency (OR)			Dietary diversity (β)		
	0–5.9 months			6–23.9 months			6–23.9 months		
	Bangladesh^a^ (*n* = 977)	Vietnam^b^ (*n* = 948)	Ethiopia^b^ (*n* = 602)	Bangladesh^a^ (*n* = 1211)	Vietnam^b^ (*n* = 1095)	Ethiopia^b^ (*n* = 870)	Bangladesh^a^ (*n* = 1211)	Vietnam^b^ (*n* = 1095)	Ethiopia^b^ (*n* = 870)
Education									
1–5 years schooling	Ref	Ref	Ref	Ref	Ref	Ref	Ref	Ref	Ref
No schooling	1.36	—	1.47	1.18	—	1.31	−0.12	—	−0.012
6–9 years schooling	1.01	0.68	2.80*	0.95	1.24	1.17	0.061	0.36*	0.057
10–12 years schooling	1.16	0.75	1.25	1.18	1.02	0.96	0.26	0.32	−0.23
College or higher	—	0.456	—	—	2.34*	—	—	0.49*	—
Knowledge	*1.22* *	1.11**	1.03	0.98	1.03	1.05	*0.17* ***	*0.14* **	*0.17* ***
Height	1.01	*1.26* *	1.0	1.02	1.02	1.01	0.0079	−0.0072	0.0067
Well‐nourished	1.60*	0.636	1.21	0.91	0.83	0.74	0.011	−0.088	0.010
Mental well‐being	*1.21* *	1.01	*1.24* *	1.01	1.02	1.02	−0.0047	0.019	0.010
Decision‐making	1.00	1.01	1.02	1.00	1.01	1.05	−0.000020	*0.13* **	0.0081
Employed	0.69	1.23	0.67	1.80*	0.84	1.27	0.0056	0.014	0.0026
Support in chores	1.03	0.967	0.96	1.00	1.02	*1.37* ***	*0.13* **	−0.0045	*0.073* *
Perceived instrumental support	0.96	0.904	0.88	0.94	0.81	0.96	0.056	0.20***	*0.080* *

*Note.* Italicized odds ratios or regression coefficients are specific to 1 *SD* higher resources for care.

a Survey included 0–47.9 months old.

b Surveys included 0–59.9 months old.

* *P* < .05.

** *P* < .01.

*** *P* < .001.

#### 3.3.2 Hygiene practices

Education was positively associated with improved drinking water and sanitation in two countries and cleanliness in all three countries (Table 4). Knowledge was positively associated with improved water in Ethiopia (OR = 1.20), improved sanitation in Vietnam (OR = 1.17), and cleanliness in Bangladesh (β = 0.13) and Ethiopia (β = 0.15). In Bangladesh, height was associated with cleanliness (β = 0.15), and well‐nourishment had associations with improved sanitation (OR = 1.34) and cleanliness (β = 0.36). Mental well‐being was positively associated with improved water in Ethiopia (OR = 1.15), improved sanitation in Bangladesh (OR = 1.20) and Vietnam (OR = 1.16), and cleanliness in all three countries. In contrast, mental well‐being had a negative association with improved sanitation in Ethiopia (OR = 0.90). Decision‐making autonomy had a positive association with cleanliness in Ethiopia (β = 0.13). Employment had a negative association with improved sanitation in Bangladesh (OR = 0.51) and cleanliness in two countries. Support in chores had a positive association with cleanliness only in Vietnam (β = 0.063). Perceived instrumental support was positively associated with improved sanitation one country and cleanliness in two countries.

**Table 4:** Adjusted associations of maternal resources for care with hygiene and health‐seeking practices

Resources for care	Hygiene practices									Health‐seeking		
	Improved drinking water (OR)			Improved sanitation (OR)			Cleanliness (β)			Immunization (OR)		
	0–47.9/0–59.9 months			0–47.9/0–59.9 months			0–47.9/0–59.9 months			12–47.9/12–59.9 months		
	Bangladesh^a^ (*n* = 4,400)	Vietnam^b^ (*n* = 4,029)	Ethiopia^b^ (*n* = 2,746)	Bangladesh^a^ (*n* = 4,400)	Vietnam^b^ (*n* = 4,029)	Ethiopia^b^ (*n* = 2,746)	Bangladesh^a^ (*n* = 4,400)	Vietnam^b^ (*n* = 4,029)	Ethiopia^a^ (*n* = 2,746)	Bangladesh^a^ (*n* = 2,983)	Vietnam^b^ (*n* = 2,607)	Ethiopia^b^ (*n* = 1,800)
Education												
1–5 years schooling	Ref	Ref	Ref	Ref	Ref	Ref	Ref	Ref	Ref	Ref	Ref	Ref
No schooling	0.79	—	0.74*	0.91	—	0.64	−0.57***	—	−0.47***	0.94	—	0.99
6–9 years schooling	1.08	1.28*	0.78	1.19	1.15	0.79	0.40***	0.25**	0.49*	1.23	1.08	1.13
10–12 years schooling	1.00	1.57**	2.80	1.72**	1.39*	0.18	0.55**	0.43***	0.64	2.54**	1.08	1.26
College or higher	—	1.19	—	—	0.69	—	—	0.59***	—	—	0.69	—
Knowledge	1.03	1.02	*1.20* **	0.99	*1.17* **	1.17	0.13***	0.011	*0.15* **	1.03	1.01	*1.20* **
Height	1.00	0.99	0.99	1.01	1.00	1.02	*0.15* ***	0.0079	0.011	1.00	1.01	0.99
Well‐ nourished	1.12	1.03	0.87	1.34*	0.99	1.75	0.36***	0.064	0.14	1.18	0.88	0.86
Mental well‐being	1.01	1.02	*1.15* *	*1.20* **	*1.16* ***	0.90*	*0.11* **	*0.071* *	*0.25* ***	0.99	1.02	0.99
Decision‐making	1.02	0.99	0.98	0.98	0.98	1.05	0.025	0.018	*0.13* *	*1.14* *	*1.49* **	0.994
Employed	1.03	1.11	0.84	0.51*	1.33	1.77	−0.35*	−0.22*	0.18	1.11	0.98	1.54**
Support in chores	0.99	1.00	1.02	1.01	1.03	1.09	0.0060	*0.063* *	0.0037	0.98	1.02	0.99
Perceived instrumental support	1.10	1.02	0.94	1.01	1.01	1.62*	0.35***	0.050	0.11*	1.23**	1.00	1.06

*Note.* Italicized odds ratios or regression coefficients are specific to 1 *SD* higher resources for care.

a Survey included 0–47.9 months old.

b Surveys included 0–59.9 months old.

* *P* < .05.

** *P* < .01.

*** *P* < .001.

#### 3.3.3 Health‐seeking practice (full immunization)

Maternal education was positively associated with child immunization in Bangladesh (10–12 years of schooling: OR = 2.54; Table 4). Knowledge (Ethiopia) and decision‐making autonomy (Bangladesh and Vietnam) were associated with immunization. Additionally, maternal employment and perceived instrumental support were positively associated with immunization in Ethiopia (OR = 1.54) and Bangladesh (OR = 1.23), respectively.

#### 3.3.4 Family care behaviours

Education was positively associated with psychosocial stimulation in Vietnam (both groups) and among older in children in Bangladesh (Table 5). Additionally, education was associated with availability of adequate caregiver of the younger children in Vietnam and Ethiopia and older children in Bangladesh and Ethiopia. Knowledge was associated with psychosocial stimulation (both groups) and availability of adequate caregiver (older children) in Vietnam. Height was positively associated with psychosocial stimulation of older children in Vietnam (β = 0.084) and availability of adequate caregiver of younger children in Bangladesh (OR = 1.51). Well‐nourishment had a positive association with psychosocial stimulation of older children in Bangladesh (β = 0.20). Mental well‐being was positively associated with psychosocial stimulation in Vietnam (older children) and availability of adequate caregiver in Vietnam (older children) and Ethiopia (younger children). Decision‐making autonomy was positively associated with older children's psychosocial stimulation in Bangladesh (β = 0.10) but had a negative association with availability of adequate caregiver of older children in Ethiopia (OR = 0.93). Employment was negatively associated with adequate care of both age groups in Bangladesh and older children in Ethiopia. Support in chores was positively associated with psychosocial stimulation in Bangladesh (younger children) and Vietnam (both age groups). In contrast, support in chores had a negative association with availability of adequate care in Ethiopia (younger children). Perceived instrumental support was positively associated with psychosocial stimulation (both age groups in Bangladesh and Vietnam) and availability of adequate care (older group in Bangladesh).

**Table 5:** Adjusted associations between maternal resources for care and family care behaviours

Resources for care	Psychosocial stimulation (β)				Adequate caregiver (OR)					
	6–23.9 months		24–47.9/24–59.9 months		6–23.9 months			24–47.9/24–59.9 months		
	Bangladesh^a^ (*n* = 1,211)	Vietnam^b^ (*n* = 1,095)	Bangladesh^a^ (*n* = 2,180)	Vietnam^b^ (*n* = 1,972)	Bangladesh^a^ (*n* = 1,211)	Vietnam^b^ (*n* = 1,095)	Ethiopia^b^ (*n* = 870)	Bangladesh^a^ (*n* = 2,180)	Vietnam^b^ (*n* = 1,972)	Ethiopia^b^ (*n* = 1,254)
Education										
1–5 years schooling	Ref	Ref	Ref	Ref	Ref	Ref	Ref	Ref	Ref	Ref
No schooling	−0.16	—	−0.14	—	1.20	—	0.77	0.64	—	0.67*
6–9 years schooling	0.13	0.067	0.28**	0.224*	2.61	4.22**	2.41*	2.93**	1.00	0.94
10–12 years schooling	0.13	0.29	0.58***	0.507***	1.14	2.02	1.32	1.06	0.93	3.78
College or higher	—	0.75***	—	1.40***	—	14.6*	—	—	1.17	—
Knowledge	−0.0039	*0.16* **	0.0034	*0.28* ***	1.01	0.97	1.05	0.98	1.10**	0.97
Height	0.011	−0.0037	−0.0038	*0.084* *	*1.51* *	1.03	1.00	0.99	1.01	1.01
Well‐nourished	0.14	−0.0064	0.20**	−0.044	0.68	0.45	0.70	0.75	0.98	1.00
Mental well‐being	0.010	−0.0061	−0.0055	*0.084* *	1.04	1.02	*1.23* *	1.04	*1.37* **	1.02
Decision‐making	0.020	0.0026	*0.10* **	−0.027	0.87	0.92	0.97	0.93	0.92	0.93**
Employed	−0.080	0.021	0.21	−0.073	0.25**	1.48	0.84	0.37**	0.36	0.74*
Support in chores	*0.098* *	*0.17* ***	0.013	*0.27* ***	0.97	1.04	0.94*	0.96	1.01	0.97
Perceived instrumental support	0.14*	0.21***	0.14**	0.12*	1.26	1.25	0.97	1.35**	0.81	1.08

*Note.* Italicized odds ratios or regression coefficients are specific to 1 SD higher resources for care.

a Survey included 0–47.9 months old.

b Surveys included 0–59.9 months old

* *P* < .05.

** *P* < .01.

*** *P* < .001.

## 4 DISCUSSION

Higher maternal education, knowledge, height, well‐nourishment, and perceived instrumental support were positively associated with better care behaviours. Mental well‐being, decision‐making, employment, and support in chores had positive associations with some types of care behaviours, but the associations were in the negative direction in some instances. Education was more consistently associated with care behaviours than other measures of resources for care. Additionally, associations between resources for care and care behaviours differed by study settings and care behaviours.

Education was positively associated with all care behaviours. Knowledge had positive associations with all care behaviours except minimum meal frequency. Mothers with higher education are more likely to have higher health and nutrition‐related knowledge, understand health education messages, be receptive to modern medicine, and be adaptive to their new roles and environment (Engle, 1999; Glewwe, 1999; Wachs, 2008). Educated mothers are also more likely to manage household resources and allocate more resources to children (Wachs, 2008). Knowledge learned outside classroom can also play a critical role in improving maternal responsiveness to children's need (Glewwe, 1999). Previous studies have found positive effects of maternal education and knowledge on complementary feeding (Monterrosa, Pelto, Frongillo, & Rasmussen, 2012; Senarath, Godakandage, Jayawickrama, Siriwardena, & Dibley, 2012), food hygiene (Guldan et al., 1993), use of closed latrines (Semba et al., 2008), and immunizations (Abuya, Onsomu, Kimani, & Moore, 2011; Semba et al., 2008).

Maternal height had positive associations with EBF, cleanliness, psychosocial stimulation, and availability of adequate caregiver. Height is the expression of genetic make‐up and the mother's childhood environment. Maternal short stature reflects economic deprivation and restricted circumstances, which may negatively affect care behaviours (Addo et al., 2013; Hernandez‐Diaz et al., 1999). Short stature has been linked with poor health, productivity, and learning, which may also have negative effect on care behaviours (Dewey & Begum, 2011).

Well‐nourishment was positively associated with EBF, improved sanitation, cleanliness, and psychosocial stimulation. A community‐based randomized trial from Guatemala showed that undernourished women who had received high energy supplements were more likely to exclusively breastfeed their infants (González‐Cossío, Habicht, Rasmussen, & Delgado, 1998). Maternal undernutrition increases morbidity, reduces activity levels and productivity, and diminishes ability to care for herself and family (Kulasekaran, 2012; Walker, 1997).

Mental health was positively associated with EBF, improved water, cleanliness, psychosocial stimulation, and availability of adequate caregiver. Mental health problems may lead to inappropriate or inadequate care such as suboptimal breastfeeding (Dennis & McQueen, 2009; McLearn, Minkovitz, Strobino, Marks, & Hou, 2006), non‐responsive feeding (Hurley, Black, Papas, & Caufield, 2008), poor psychosocial stimulation (Black et al., 2007; McLearn et al., 2006; Murray, Cooper, & Hipwell, 2003), and inadequate health‐seeking including immunization uptake (Minkovitz et al., 2005). Women with poor mental health are likely to live in adverse environments, be withdrawn from the surrounding, and have lower support system, self‐esteem, and confidence, which may result in less time and capacity to perform care behaviours (Patel, Rahman, Jacob, & Hughes, 2004). Poor mental health is also linked with physical symptoms, fatigue, poor health‐seeking practices, and involvement in high‐risk behaviours that may compromise care behaviours (Black et al., 2007; Patel et al., 2004). Additionally, poor mental health may negatively influence cognitive functioning, memory, and learning, which may increase the likelihood of inappropriate care behaviours (Austin, Mitchell, & Goodwin, 2001). In this study, mental well‐being was positively associated with improved sanitation in Bangladesh and Vietnam, but the association was negative in Ethiopia. In Ethiopia, improved sanitation could be influenced by factors other than those related to mother or family like sanitation projects (O'loughlin, Fentie, Flannery, & Emerson, 2006). Additionally, sanitation has been included in agendas of heath sectors and government in the country. There may also be lack of awareness about benefits of improved sanitation in this setting (Kumie & Ali, 2005). Future studies are needed to examine the reasons for the negative association of maternal mental well‐being with improved sanitation.

Decision‐making autonomy had positive associations with dietary diversity, cleanliness, immunization, and psychosocial stimulation. In contrast, decision‐making autonomy was negatively associated with availability of adequate caregiver. Consistent with our findings, previous research shows mixed influence of maternal autonomy. A study from Nicaragua found that children of women who had middle level of autonomy had better complementary feeding practices, and children of women with lowest autonomy had improved breastfeeding practices (Ziaei et al., 2015). Autonomy allows mothers to make decisions in favour of children, and they are more likely to spend on health and nutrition (Quisumbing & Maluccio, 2000; Thomas, 1990). On the other hand, autonomy may equip mothers with freedom to move, work, and visit people, which may result in leaving children alone or with minors.

Employment had positive associations with minimum meal frequency and immunization but had negative associations with improved sanitation, cleanliness, and availability of adequate caregiver. Employment may improve economic capacity to spend in complementary feeding and health care; on the other hand, it may impede care behaviours, especially those that are time‐consuming and labour‐intensive. Employed mothers are more likely to leave children alone or with minors (Engle et al., 1999). Children are at higher risk of poor care if mothers are involved in jobs that are low‐paying, informal, and with poor‐working conditions (Engle et al., 1999).

Support in chores was positively associated with minimum meal frequency, dietary diversity, cleanliness, and psychosocial stimulation. Perceived instrumental support was positively associated with dietary diversity, improved sanitation, cleanliness, immunization, psychosocial stimulation, and availability of adequate caregiver. Mothers often have time conflict between childcare and household chores, especially in settings where gender norms related to childcare and household chores are prominent (Nakahara et al., 2006). Both employed and not employed women work long hours on domestic tasks and care for household members, which may leave women with “time poverty” or less time for leisure activities (Engle et al., 1999; Nakahara et al., 2006; Warren, 2003). Support in chores provides help with care of children (for example, feeding and bathing). Support in chores also reduces mother's responsibilities, preserves maternal time and energy levels, and consequently enables mothers to provide better care (Nakahara et al., 2006). Perceived support including perceived instrumental support among parents may affect parenting practices and child outcomes by reducing stress (Taylor, Conger, Robins, & Widaman, 2015). Perception of instrumental support may improve economic security and well‐being of parents. Turney found that perception of instrumental support had positive associations with overall health of child even after accounting for maternal and child characteristics (Turney, 2013). Additionally, social resources may buffer and ameliorate negative effects of adversities like poverty (Evans, Boxhill, & Pinkava, 2008). Support from family and friends is positively related with optimal maternal behaviours such as more praising of children and being less intrusively controlling, less punitive parental attitude, improved maternal–child interaction, and provision of more stimulating environment (Burchinal, Follmer, & Bryant, 1996; Jennings, Stagg, & Connors, 1991; McCurdy, 2005); however, all aspects of social support may not be beneficial (Antonucci, Akiyama, & Lansford, 1998). Support in chores had a negative association with availability of adequate caregiver of younger children in Ethiopia. The available family and friends may be engaged in helping with other chores; therefore, children may be left alone or with minors. Additionally, social relations and support may increase demand and add stress, which may compromise care to children (Black & Nitz, 1996; Uchino, Cacioppo, & Kiecolt‐Glaser, 1996).

Associations of resources for care with care behaviours differed across study settings. For example, improved maternal knowledge, well‐nourishment, and mental well‐being had positive associations with EBF in Bangladesh; higher knowledge and height had positive associations with EBF in Vietnam, and maternal education and mental well‐being had positive associations with EBF in Ethiopia. Despite these differences across study settings, our study suggests that improvement in status of mothers likely will translate into better care received by children. The differences across study settings may be because care behaviours may be affected by attributes other than those of mothers and families such as government policies, health system, and the private sector (Britto et al., 2017; Maggi et al., 2010; Nguyen, Menon, et al., 2014). Contextual factors such as ethnicity, culture, neighbourhood, and community shape parenting beliefs and practices (Kotchick & Forehand, 2002). A study by Wiysonge and colleagues also found that care received by children is shaped by the community and country‐level factors. Unimmunized children were more likely to be from urban areas, communities with higher illiteracy rates, and countries with higher fertility rates (Wiysonge, Uthman, Ndumbe, & Hussey, 2012). These contextual factors also influence the resources available to mothers. For example, a cross‐country study found that women's participation in labour force is affected by the attitude of men towards the female labour force participation. Women were more likely to work if men in their country had a favourable attitude towards it (Antecol, 2003).

Our study highlights that associations of resources for care with care behaviours depend on type of care behaviours, which has been found in previous studies as well (Guldan et al., 1993; Peter & Kumar, 2014). In some instances, the associations of resources for care with family care behaviours differed between younger and older children. The trend of positive association between resources for care and psychosocial stimulation was seen more among older children than younger ones. This trend could be due to belief that younger children may not need psychosocial stimulation like older ones. Rapid brain development occurs during the early period of life, and psychosocial stimulation to the young children is very essential to ensure the optimal early child development (Aboud & Yousafzai, 2015; Brown & Pollitt, 1996). Our findings warrant consideration of interventions that increase awareness about the need of psychosocial stimulation among the younger children. This study included multiple settings that allowed us to compare the findings in three different contexts: and increased the generalizability, especially for low‐ and middle‐income countries. Vietnam had lower EBF but higher improved complementary feeding, and Ethiopia had higher EBF but lower improved complementary feeding. The study setting in Vietnam was more materialistically advanced than Bangladesh and Ethiopia. Our analyses are based on large sample size and inclusion of multiple categories of resources for care and multiple care behaviours. Data on some variables were based on self‐reporting, but to improve data quality, training was provided to the data collector. Reliable and validated instruments were used for data collection (for example, SRQ‐20). Furthermore, to collect data on IYCF practices, mothers were asked if certain foods and liquids were consumed by the children in the past 24 hr rather than asking direct questions about children's feeding practices. Additionally, the cross‐sectional nature of the data does not allow us to draw causal inferences. Although we controlled for variables at household, parents, and child level, resources for care and care behaviours can be influenced by other conditions as well like father's knowledge and belief, or community social norms (Taylor, Hamvas, Rice, Newman, & DeJong, 2011; Yee & Oh, 2006). Furthermore, there may be joint influence of causation and selection (Miech, Caspi, Moffitt, Wright, & Silva, 1999). For example, resources for care may improve hygiene practices that in turn may enhance resources for care such as physical and mental health.

In conclusion, maternal education and knowledge, physical health, mental well‐being, autonomy, workload, and social support had positive associations with care behaviours, but in a few instances, the associations were in a negative direction. Resources available to mothers may influence the provision of appropriate IYCF, hygiene, health‐seeking, and family care. Interventions that aim to improve maternal education, knowledge, health, autonomy, and support system are likely to have positive effects on care received by children. Interventions that aim to improve women' status need to focus not only on women but also on their family and community. Additionally, provision of nurturing environment during in utero and childhood period may help in achieving resources like optimal height. Future studies that examine the association of other types of resources for care (for example, emotional support) with care behaviours may help to further understand the role of resources for care in improving care. Qualitative studies may be helpful to understand the differences in the findings across countries. Additionally, longitudinal studies may provide evidence on causal associations. The findings of this study reinforce that the provision of resources to mothers are essential to improve care behaviours.

## ACKNOWLEDGMENTS

This study was funded by the Bill & Melinda Gates Foundation, through Alive & Thrive, managed by FHI 360, and the Patrice L. Engle Dissertation Grant in Global Early Child Development. Additional financial support to the evaluation study was provided by the CGIAR Research Program on Agriculture for Nutrition and Health (A4NH), led by the International Food Policy Research Institute.

## CONFLICTS OF INTEREST

The authors declare that they have no conflicts of interest.

## CONTRIBUTIONS

SB conducted the data analysis and wrote the paper under the guidance of EAF and PHN. SM and MA collaborated in the interpretation of results and critically reviewed the manuscript.
